# Isolation and identification of hoylesella marshii causing pleural infection: a case report

**DOI:** 10.1186/s12879-024-09586-5

**Published:** 2024-07-08

**Authors:** Hairong Zhang, Man Li, Sen Wang

**Affiliations:** 1 Department of Clinical Laboratory Medicine, Nanjing Drum Tower Hospital, Affiliated Hospital of Medical School, Nanjing University, Nanjing, 210008 China; 2 Department of Clinical Laboratory Medicine, Yancheng Tinghu District People’s Hospital, Yancheng, 224001 China

**Keywords:** Hoylesella marshii, Pleural infection, Contaminant, Obligate anaerobe, Isolation and identification

## Abstract

**Background:**

*Hoylesella marshii* can be isolated from human oral cavities affected by dental pulp and periodontal infections, as well as from the dental plaque of healthy individuals, making it a common bacterium within the oral microbiota. However, its role in causing pleural infections in humans is rare.

**Case presentation:**

A case of purulent pleural effusion occurred shortly after discharge in an elderly patient who had undergone surgery for gastric cancer. The infection was identified as being caused by an obligate anaerobe through laboratory culture, and was further identified as *Hoylesella marshii* causing pleural infection through 16 S rRNA gene sequence analysis. Susceptibility testing guided precise treatment with cefoperazone-sulbactam and metronidazole. The patient’s clinical symptoms improved rapidly, laboratory test indicators gradually returned to normal, and the patient ultimately recovered.

**Conclusion:**

*Hoylesella marshii* can cause pleural infections in humans. Clinical microbiology laboratories should pay special attention to the cultivation of obligate anaerobes when routine aerobic cultures do not show bacterial growth but bacteria are visible on smear staining, and when conventional identification methods fail to identify the bacterium, analysis based on the highly conserved 16 S rRNA gene sequence can accurately and specifically identify the bacterium, guiding clinicians in formulating precise anti-infection strategies.

## Introduction

*Hoylesella marshii*, originally known as *Prevotella marshii*, was first isolated from the human oral cavity and named in honor of British microbiologist Philip Marsh to commemorate his contributions to oral microbiology, as mentioned by Downes et al. in 2005 [1]. The genus *Hoylesella*, named after contemporary British gut microbiologist Lesley Hoyles, represents a reclassification of species within the genus *Prevotella* into one of seven new genera [2]. The genus *Prevotella*, proposed by Shah and Collins in 1990 [3], was differentiated from the genus Bacteroides and comprises a group of Gram-negative, obligate anaerobic, non-motile, non-spore-forming rods [4]. *Prevotella* is a predominant bacterium in the human oral microbiota, involved in nearly all oral infections and frequently found in infections of the head, neck, and thoracic cavity, making it an important opportunistic pathogen [5]. Infections caused by *Prevotella* are more likely to occur in individuals with weakened immune systems, such as those with chronic diseases and the elderly.

There have been no reported cases of thoracic infections caused by *Hoylesella marshii*. Here, we report a case of a thoracic infection caused by *Hoylesella marshii*, detailing the isolation, identification, and antimicrobial susceptibility of the bacterium, and provide a summary and reflection on the case.

## Case presentation

### Medical history

**Fig. 1:**
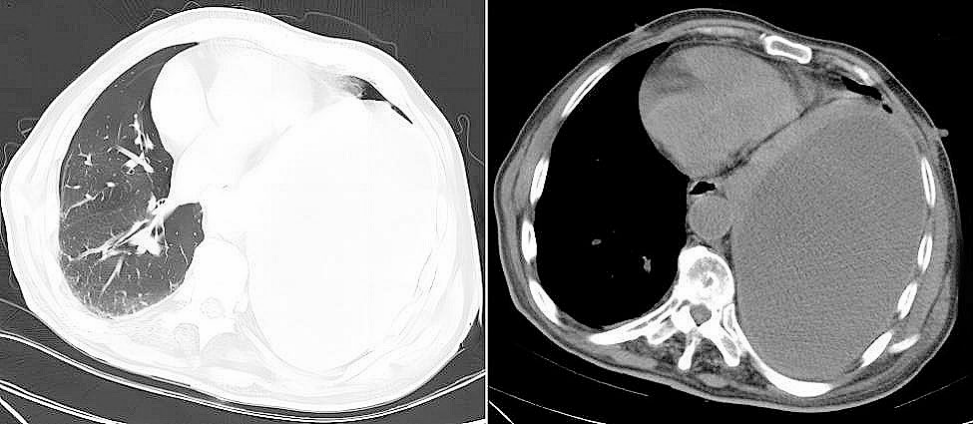
CT imaging findings before patient treatment

The patient is a 74-year-old male who presented with symptoms of chest tightness, dyspnea after activity, and occasional left chest pain without any apparent cause about one month ago. The patient also experienced occasional coughing but no chills or fever, and the symptoms of chest tightness and dyspnea would alleviate after rest. At that time, these symptoms were not given due attention or treated. Later, the symptoms of chest tightness and dyspnea after activity worsened and could not be relieved, and in recent days, the pain in the left chest has significantly intensified. Consequently, the patient was brought to our hospital’s emergency department by his family, where an emergency CT scan revealed encapsulated pleural effusion in the left thoracic cavity and atelectasis of the left lung, as shown in Fig. 1. The preliminary diagnosis was pleural effusion, and the patient was admitted to the hospital. The patient had poor dietary intake over the past two months. Upon admission, the patient was conscious but appeared in pain and experienced difficulty breathing. He had undergone surgery for gastric cancer at a hospital in Shanghai one and a half months prior.

### Physical examination upon admission

The patient’s temperature was 37.2 °C, respiratory rate was 26 breaths per minute, and pulse was 109 beats per minute. There was mild cyanosis of the lips. The breath sounds were coarse bilaterally, with significantly weakened respiratory movements and breath sounds on the left side compared to the right. A few scattered fine wet rales could be heard.

### Laboratory examinations

#### Hematological tests upon admission

White Blood Cells (WBC): 5.87 × 10^9^/L, Neutrophils (N): 87%; Procalcitonin (PCT): 0.0742 ng/mL, Interleukin-6 (IL-6): 0.3054 ng/L; Arterial Blood Gas Analysis showed a Partial Pressure of Oxygen (PO2) of 54.59 mmHg, pH of 7.50, Actual Bicarbonate Ion (HCO3) of 30.04 mmol/L, and Potassium (K^+^) of 3.08 mmol/L.

#### Pleural effusion analysis

The pleural effusion appeared as a grayish-white, turbid, purulent fluid. The WBC count in the pleural fluid was 1,473 × 10^6^/L with 91% of the cells being segmented neutrophils. The Rivalta test was strongly positive.

#### Microbiological examination of pleural effusion

**Fig. 2:**
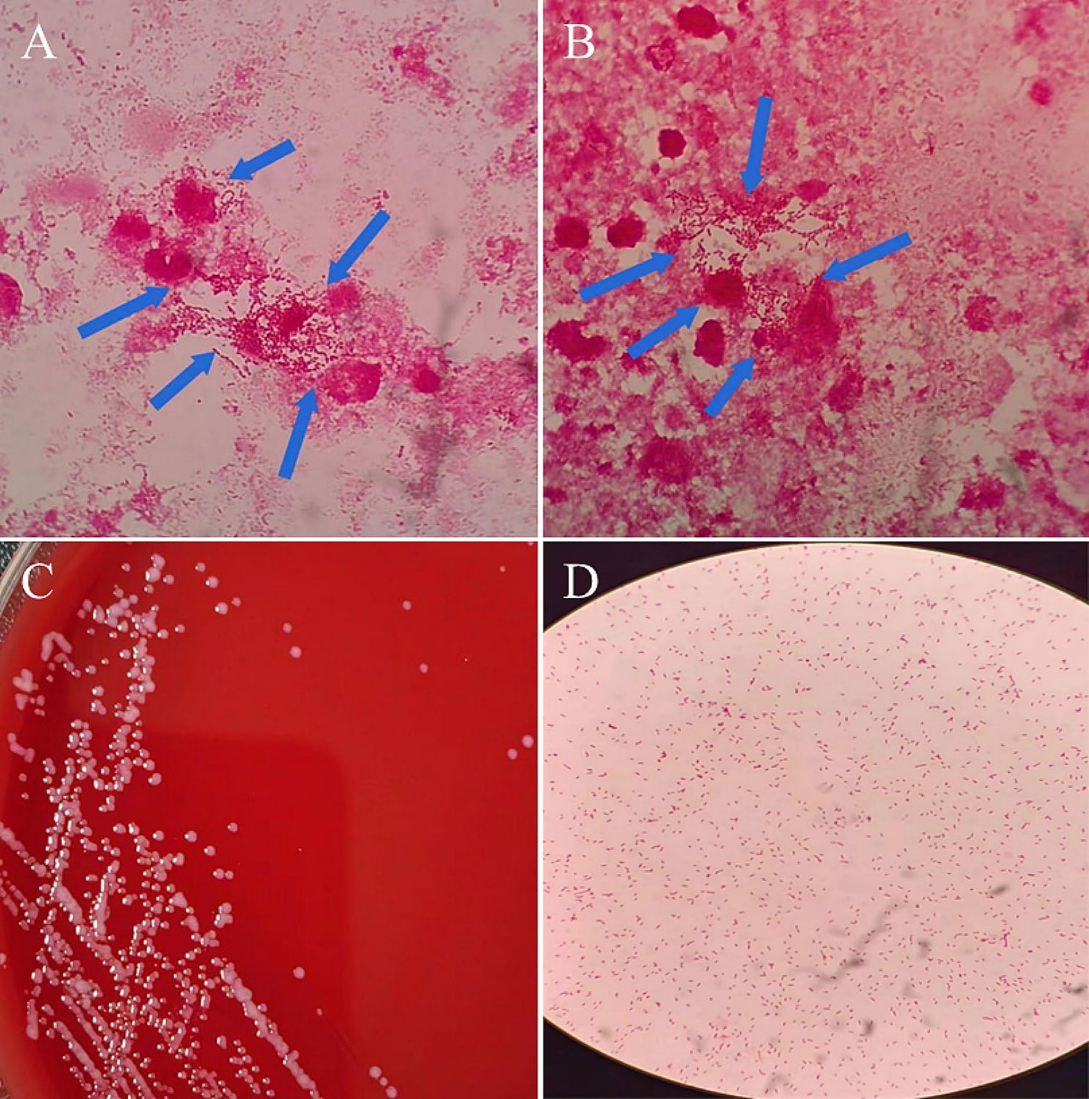
Bacterial smear staining and bacterial culture

In a biosafety cabinet, the specimen was prepared for smear and fixation. Gram staining and oil immersion microscopy revealed a large number of Gram-negative rods and inflammatory cells, as illustrated in Fig. 2A and B. The specimen was inoculated onto Columbia blood agar, chocolate agar, and MacConkey agar plates within a sterile environment, and then placed in an incubator containing 5% CO_2_ at 35 °C for routine aerobic culture for 24 and 48 h. No bacterial growth was observed on any of the plates after this period. Observing no bacterial growth after 24 h of aerobic culture, the laboratory immediately communicated with the clinical physicians, indicating that the bacteria seen under the microscope could be obligate anaerobes. The specimen was then inoculated onto Columbia blood agar, chocolate agar, and MacConkey agar plates and placed inside a GENbag anaerobic bag (Bio-Mérieux, France) for anaerobic culture in an incubator containing 5% CO_2_ at 35 °C. After 48 h of culture, bacterial growth was observed on the blood agar plate but not on the chocolate and MacConkey agar plates. The colonies on the blood agar were milky white, moist, slightly raised, round with neat edges, approximately 0.2 mm in diameter, and showed no hemolysis, as depicted in Fig. 2C. Gram staining of colonies from the anaerobic culture revealed small Gram-negative rods, dispersed individually, as shown in Fig. 2D. Mass spectrometry identification was performed using a Bio-Mérieux spectrometer; a small amount of the bacterial colony was smeared on a target plate, covered with matrix solution, and dried before undergoing laser scanning. The test plate hole and the ATCC 8739 E. coli quality control hole both turned green, indicating a pass. Peaks of the bacterial laser scan were present in the spectrometer, but no final identification result was obtained (as the bacterium was not included in the spectrometer’s database). Subsequently, the bacterium was sent to a third-party sequencing company (Shanghai Map Biotech Co., Ltd.) for 16 S rRNA gene sequencing. Identified as *Hoylesella marshii* by the 3730XL DNA Analyzer, this bacterium is an obligate anaerobe, with a sequencing identification reliability of 99.6%.

#### Antimicrobial susceptibility testing

**Fig. 3:**
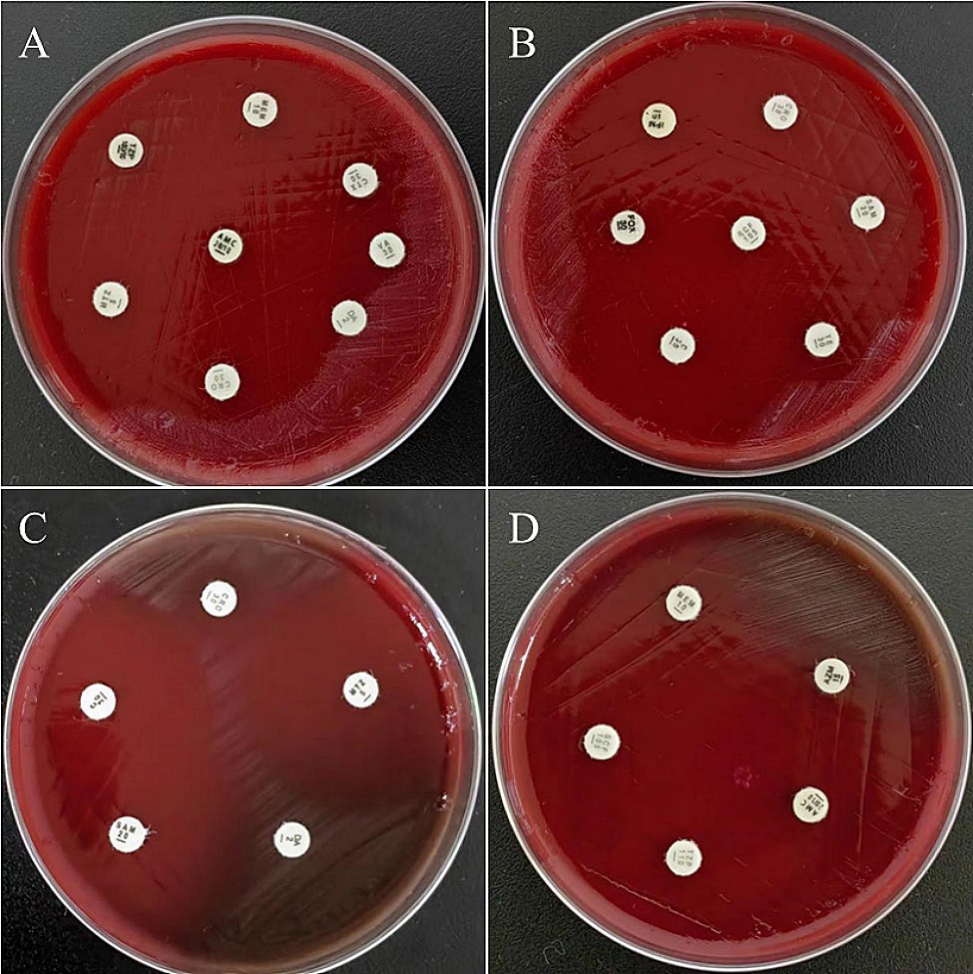
Antimicrobial Susceptibility Testing

**Fig. 4:**
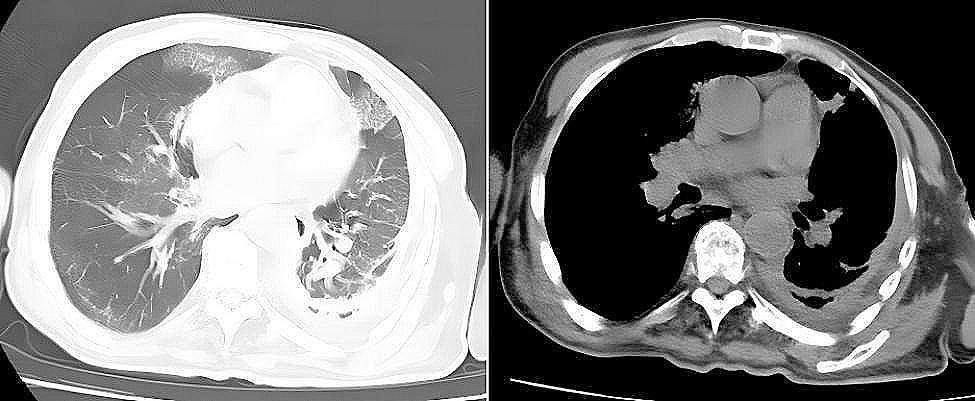
CT imaging findings post-treatment

Following the standards for antimicrobial susceptibility testing as outlined by the Clinical and Laboratory Standards Institute (CLSI) M100 ED33, a selection of antimicrobial agents effective against obligate anaerobic Gram-negative rods was tested. These agents included Amoxicillin-Clavulanic Acid (AMC), Ampicillin-Sulbactam (SAM), Piperacillin-Tazobactam (TZP), Cefoperazone-Sulbactam (SCF), Meropenem (MEM), Imipenem (IPM), Metronidazole (MTZ), Chloramphenicol (C), Clindamycin (DA), Tetracycline (TE), Cefoxitin (FOX), Ceftriaxone (CRO), and Tobramycin (TOB). The disk diffusion method was used to evaluate the sensitivity of the antimicrobial agents. The bacterial suspension was adjusted to a 0.5 McFarland standard and inoculated onto Columbia blood agar plates using the spread plate method. The antimicrobial susceptibility testing plates were then placed in an anaerobic environment (GENbag anaerobic bag, produced by Bio-Mérieux, France) with Thermo anaerobic indicators, and incubated at 35 °C for 48 h. Given the lack of reference ranges for disk diffusion method for obligate anaerobes, a zone diameter of 6 mm was considered resistant, though this may underestimate the true incidence of resistance but provides a definitive boundary for clear interpretation. A diameter of ≤ 30 mm indicated reduced sensitivity. The susceptibility results showed that *Hoylesella marshii* was sensitive to SCF (48 mm), MTZ (40 mm), AMC (50 mm), SAM (39 mm), TZP (43 mm), IPM (38 mm), and C (38 mm); whereas it was resistant or showed reduced sensitivity to DA (6 mm), TOB (6 mm), CRO (16 mm), TE (13 mm), MEM (19 mm), and FOX (20 mm), as shown in Fig. 3A–D. Based on the results of the antimicrobial susceptibility testing, the clinicians promptly adjusted the medication regimen to Ornidazole and Cefoperazone-Sulbactam, combined with closed drainage of the pleural effusion. The patient’s bacterial infection inflammation markers, including Procalcitonin (PCT: 0.0328 ng/mL) and the percentage of Neutrophils (N%: 43%), continued to decrease. Imaging studies also showed improvement, and clinical symptoms were getting better. One week after adjusting the medication, a follow-up CT scan revealed that the pleural effusion in the left thoracic cavity had been essentially absorbed, as depicted in Fig. 4. The patient ultimately recovered and was discharged from the hospital.

## Discussion and conclusions

*Prevotella* is a Gram-negative obligate anaerobic bacterium that constitutes an important part of the human microbiota, present in multiple parts of the body, and is also an important opportunistic pathogen. Patients with conditions such as primary or acquired immunodeficiencies, organ transplants, and malignancies are at a high risk of infection by this bacterium. Additionally, specific populations like the elderly, children, and pregnant women, who may have lowered immunity, are also more susceptible to infection. *Hoylesella marshii* (*Prevotella marshii*), initially isolated from the human oral cavity [1], has been found to increase the abundance of the genus *Prevotella*, which is not only associated with Th17-mediated mucosal inflammation but also promotes mucosal Th17 immune responses and the recruitment of neutrophils, thus exacerbating chronic inflammation, according to Larsen [6]. Said et al. reported that obligate anaerobic *Prevotella* species are associated with mucosal inflammatory responses [7].

*Prevotella* is commonly isolated from the oral cavity, upper respiratory tract, or urogenital tract, and it is most prevalent in the human mouth [2]. It is also an important member of the mammalian gut microbiome, with many species having been isolated [8]. *Hoylesella marshii* can be isolated from dental pulp and periodontal infections in the human oral cavity, as well as from the dental plaque of healthy individuals [1]. Generally, oral commensal bacteria are usually adapted to the specific environment of the oral cavity, including humidity, pH, and nutrients. The environment of the lungs and pleural cavity is significantly different from that of the oral cavity, making it difficult for these bacteria to survive and reproduce. The lungs and pleural cavity have robust defense mechanisms, including ciliary movement, mucus secretion, and the presence of immune cells. These factors and defense mechanisms can effectively prevent the invasion and colonization of foreign pathogens, making it difficult for oral commensal bacteria to cause thoracic or pulmonary infections [9]. Currently, there are no reports of *Hoylesella marshii* causing lung or thoracic infections.

In this case, the pleural effusion sample was obtained through percutaneous puncture for closed pleural drainage on the second day after the patient’s admission, minimizing the risk of bacterial contamination from prolonged catheterization. The sample underwent routine aerobic culture and Gram staining simultaneously, revealing a large number of white blood cells and Gram-negative rods in “combat,” with clusters of white cells enveloping groups of bacteria and many white cells appearing fragmented. The human immune response to invasive infections involves chemotaxis, encapsulation (infiltration), phagocytosis, and digestion of white blood cells. To confirm whether the bacterium was an infectious agent rather than a contaminant, it was necessary to observe the “battle” between white blood cells and microorganisms to accurately identify the true “enemy.” Therefore, it was concluded that the bacterium was pathogenic rather than a contaminant. Since obligate anaerobes do not grow in routine aerobic cultures, which can easily miss such bacteria, the purulent nature of the patient’s sample, the anaerobic characteristics of the infection site, and the results of the general bacterial smear test prompted proactive and timely communication with the clinical doctors. It was determined that the pleural effusion was caused by an obligate anaerobic bacterial infection, providing a basis for precise anti-infective treatment promptly. Clinically, from the moment the patient was admitted, bacterial infection was highly suspected based on clinical features, and empirical anti-infective treatment with Cefuroxime was initiated, but the treatment was not effective. After 24 h of aerobic culture with no bacterial growth observed, the sample was subjected to anaerobic culture, resulting in the growth and ultimate identification of *Hoylesella marshii* (*Prevotella marshii*).

Due to the rarity of thoracic infections caused by *Hoylesella marshii*, there is currently a lack of unified standards for the treatment of this *Prevotella marshii* bacterium. Additionally, the Clinical and Laboratory Standards Institute (CLSI) M100 ED33 standards for antimicrobial susceptibility testing do not provide reference ranges for the disk diffusion method for obligate anaerobes, which poses many inconveniences for clinical practice. One study outlined the correlation between *Prevotella* spp. carrying the tetQ resistance gene (with zone diameters of 12–20 mm) and reduced susceptibility to Doxycycline (DOX) as 100% [10]. Another study reported that 83% of tetQ resistance gene-positive isolates showed intermediate or complete resistance to Tetracycline, indicating that tetQ is a reliable marker for reduced susceptibility to Tetracycline antibiotics. A median zone diameter of 12 mm was observed in strains carrying the cfxA3 beta-lactamase gene, compared to 32 mm in strains without cfxA3 [11]. The cfxA type beta-lactamase gene represents the most significant resistance mechanism in clinical isolates of *Prevotella* spp [12]. Clearly, there is a high correlation between the zone diameters of antimicrobial drugs and their susceptibility.

This study reports a case of chest infection caused by *Hoylesella marshii*. *Hoylesella marshii* is an anaerobic Gram-negative bacterium and a known member of the oral microbiome. In cases of tracheal intubation, immunosuppression, or severe oral infections, oral bacteria can potentially spread to the lungs or thoracic cavity [13, 14]. The patient in this case was elderly, and shortly after undergoing gastric cancer surgery, the patient’s immune function declined. The ectopic growth of *Hoylesella marshii*, as an opportunistic pathogen, might have been the cause of the infection. Many oral commensal or pathogenic bacteria can indeed cause infections in other parts of the body, but they are usually considered only after other pathogens have been excluded, which can lead to delays in treatment. Therefore, this study suggests that for postoperative patients or those with weakened immune systems, the possibility of oral anaerobic bacterial infections should be considered early when lung or thoracic infections occur. When ectopic infections by bacteria such as *Hoylesella marshii* or *Prevotella* are suspected, it is necessary to communicate promptly with clinical teams and thoroughly analyze the patient’s medical history to determine the possible routes of infection. The 16 S rRNA gene sequence analysis, based on highly conserved sequences, can be used for the rapid identification of clinical isolates of this genus, and targeted treatment should be administered based on the identified pathogens.

## Data Availability

Not applicable.
